# A novel biomarker, active whole skeletal total lesion glycolysis (WS-TLG), as a quantitative method to measure bone metastatic activity in breast cancer patients

**DOI:** 10.1007/s12149-019-01359-4

**Published:** 2019-04-13

**Authors:** Mitsuru Koizumi, Kazuki Motegi, Takuro Umeda

**Affiliations:** Department of Nuclear Medicine, Cancer Institute Hospital, 3-8-31 Ariake, Koto-ku, Tokyo, 135-8555 Japan

**Keywords:** Breast cancer, Skeletal metastasis, FDG–PET/CT, Bone tumor burden, TLG

## Abstract

**Objective:**

There is no good response evaluation method for skeletal metastasis. We aimed to develop a novel quantitative method to evaluate the response of skeletal metastasis, especially lytic lesions, for treatment.

**Methods:**

A method to measure active bone metastatic burden quantitatively using F-18 fluorodeoxyglucose positron emission tomography with computed tomography (FDG–PET/CT) in breast cancer patients, whole skeletal total lesion glycolysis (WS-TLG), a summation of each skeletal lesion’s TLG, was developed. To identify active bone lesions, a tentative cutoff value was decided using FDG–PET/CT in 85 breast cancer patients without skeletal metastasis and 35 with skeletal metastasis by changing the cutoff value. Then, the WS-TLG method was evaluated by comparing to PET Response Criteria in Solid Tumor (PERCIST) or European Organization for Research and Treatment of Cancer (EORTC) criteria for only bone in 15 breast cancer patients with skeletal metastasis who were treated.

**Results:**

A cutoff value of the standardized uptake value (SUV) = 4.0 gave 91% (77/85) specificity and 97% (34/35) sensitivity. We decided on SUV = 4.0 as a tentative cutoff value. Skeletal metastases of lytic and mixed types showed higher WS-TLG values than those of blastic or intertrabecular types, although statistical significance was not tested. All 15 patients showed agreement with PERCIST or EORTC in the therapeutic bone response.

**Conclusion:**

This quantitative WS-TLG method appears to be a good biomarker to evaluate skeletal metastasis in breast cancer patients, especially lytic or mixed types. Further clinical studies are warranted to assess the clinical values of this new WS-TLG method.

## Introduction

Breast cancer often metastasizes to the skeleton [1]. Many diagnostic techniques have been employed to detect skeletal metastases and to measure their therapeutic effect [2]. Historically, x-ray has been used, but it has extremely low detectability. X-ray computed tomography (CT) has higher detectability than x-ray but lower detectability than modern techniques such as magnetic resonance imaging (MRI) and positron emission tomography (PET) using F-18 fluorodeoxyglucose (FDG) or sodium F-18 fluoride (NaF). MRI and PET are now the best techniques to detect skeletal metastases [3]. Another approach to measure bone metastatic burden is to use serum or urine bone metabolic markers [4]. Bone scintigraphy with the aid of artificial intelligence (AI) (Exni-bone or Bone-Navi) is a method to quantitate osteoblastic activity using planar images. This method is clinically successful in osteoblastic metastases such as prostate cancer [5–8]. However, an accurate quantitative method to measure bone metastatic burden in breast cancer patients has not been established.

A method to measure bone metastatic activity using sodium fluoride (NaF) PET/CT in prostate cancer has been reported [9]. We adopted this method to bone scintigraphy with single photon emission tomography with CT (SPECT/CT) in prostate cancer [10].

These tomographic methods are useful to measure osteoblastic osseous metastasis, however, are not useful to measure osteolytic osseous metastasis.

FDG uptake by osteolytic lesions is a good marker to measure [2, 11, 12], and FDG–PET/CT is now one of the main methodologies to detect and to measure skeletal metastasis.

Therefore, we tried to develop a method to measure active bone metastatic burden quantitatively using FDG–PET/CT in breast cancer patients. Briefly, (1) bone element is identified by CT attenuation. (2) Increased FDG uptake areas in the bone element are identified using a cutoff level, and these areas are regarded as the bone lesions. (3) Then, the product of lesion volume and mean FDG uptake in the lesion is calculated. This is the active bone metastatic index of a lesion: total lesion glycolysis (TLG) of one lesion. (4) The sum of TLGs of all skeletal lesions is performed; this is the active whole skeletal total lesion glycolysis (WS-TLG) of the study. This active WS-TLG method quantifies osseous tumor burden (FDG avidity and bone tumor volume).

## Patients and methods

### Patients

This is a retrospective observational study. Local ethical approval of this study was obtained (2018–1051), and informed consent was waived. All patients were diagnosed and treated for breast cancer at Cancer Institute Hospital, Ariake, Koto-ku, Tokyo, Japan.

After the local ethical approval was obtained, patient recruitment was started. Three groups of patients were selected retrospectively using our FDG–PET/CT study log.

First: eighty-five breast cancer patients without skeletal metastasis were collected from February 2018 to April 2018. The threshold or cutoff level was determined based on their data.

Second: thirty-five breast cancer patients with newly diagnosed skeletal metastasis were collected from November 2014 to April 2018. The sensitivity was calculated using FDG–PET/CT data of these patients.

Third: fifteen breast cancer patients with newly developed skeletal metastasis and at least 2 or more follow-up FDG–PET/CT studies from July 2013 to October 2018 were included.

The diagnosis of skeletal metastasis was established as follows: when the findings of FDG and CT were typical for skeletal metastasis (multiple intraosseous lesions, FDG avid, and obvious CT morphologic changes), the clinical diagnosis was established. When the patients had an intertrabecular type bone lesion or solitary osseous lesion and MRI showed the positive findings, the clinical diagnosis was established. For the patients whom the clinical diagnosis was not established even by MRI, bone biopsies were performed. The positive bone biopsy result meant the pathological diagnosis of skeletal metastasis. The patients with negative results of bone biopsy or lesions not suitable for bone biopsy underwent further follow-up studies, and the results showed the diagnosis of typical skeletal metastasis. The patients who did not have skeletal metastasis were diagnosed by normal bone FDG and CT findings and clinical follow-up for more than 6 months.

Thirty-five patients were diagnosed with skeletal metastasis. The diagnosis was established by FDG and CT in 27 of the 35 patients, by MRI in 2 patients, and by bone biopsy in 4 patients, and 2 patients were diagnosed by follow-up studies after biopsy failure.

### FDG–PET/CT study

We used routinely performed FDG–PET/CT studies for this investigation. Patients fasted for at least 6 h before being injected with 4 MBq/kg FDG and then whole-body image acquisition started at 60 min later from the top of the skull to the mid-thigh using a Discovery 610 PET/CT scanner (GE, USA) or Discovery IQ PET/CT scanner (GE, USA). Emission data were acquired for 1–3 min per bed position. Discovery 610 PET images were reconstructed using three-dimensional ordered-subsets expectation–maximization with point-spread function (OSEM + PSF); 3 iterations, 16 subsets with a 4-mm Gaussian filter, a 192 × 192 matrix (2.6 mm/pixel). Discovery IQ PET images were reconstructed using three-dimensional ordered-subsets expectation–maximization with point-spread function (OSEM + PSF); 4 iterations, 12 subsets with a 4-mm Gaussian filter, a 192 × 192 matrix (2.6 mm/pixel).

Whole-body CT scanning proceeded under the following parameters: 120 kV; auto exposure control system (noise level: SD 10); 512 × 512 matrix; beam pitch, 3.75 mm × 16-row mode.

### Active whole skeletal total lesion glycolysis (WS-TLG)

We considered that following equation gave active bone tumor burden or active WS-TLG: first, bone component was extracted by CT. A CT attenuation above 152 Hounsfield Units (HU) was regarded as the bone component [13]. Metal artifact areas and calcified areas were also included; therefore, these areas were deleted manually. Then the following formula was used to calculate active WS-TLG:

Active WS-TLG = ∑ (volume above threshold, mL) *x* (mean standardized uptake value of the lesion).

**Fig. 1:**
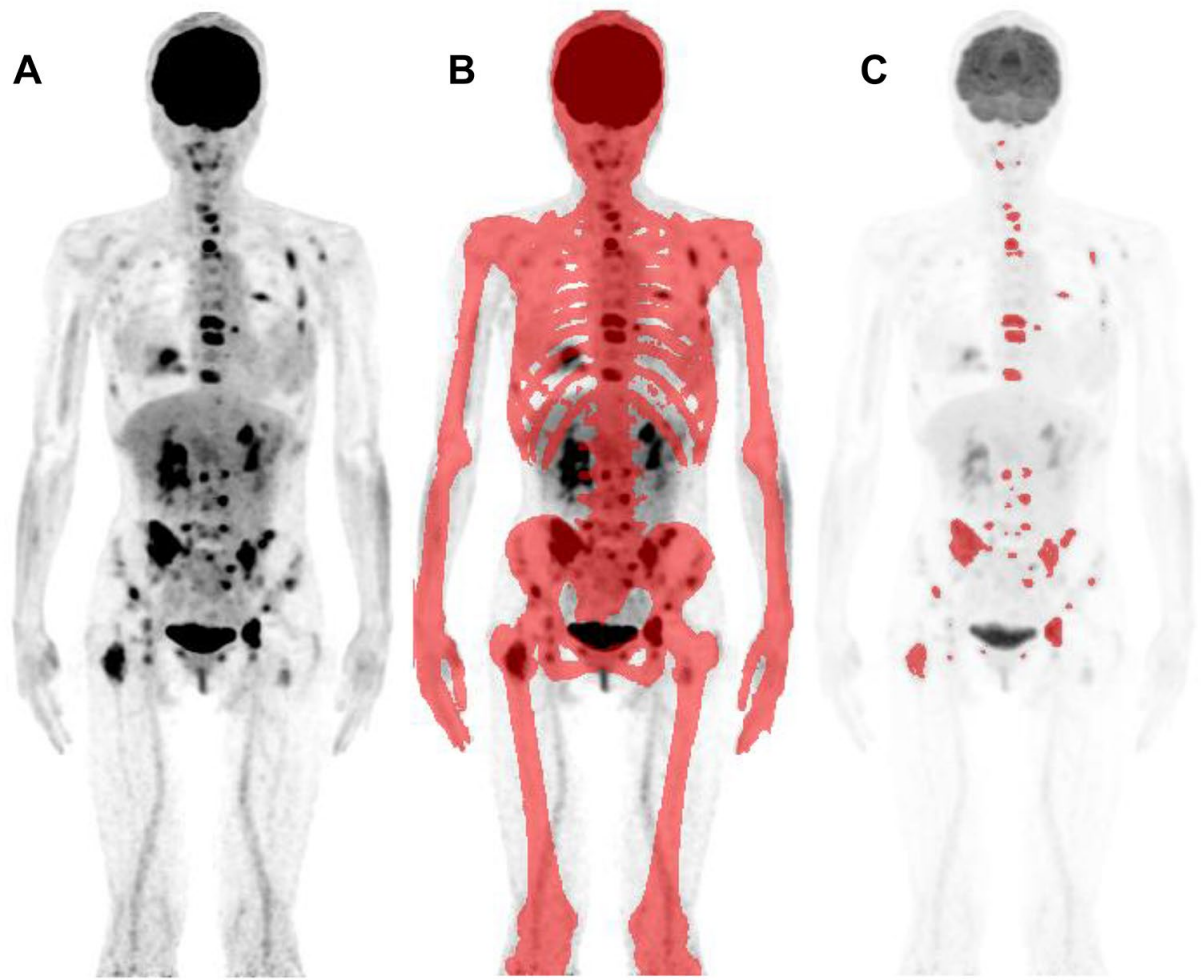
The process to obtain whole skeletal total lesion glycolysis (WS-TLG) was shown. Whole-body FDG–PET image (anterior view of maximum intensity projection; MIP) was taken (**a**). Bone areas were decided using CT attenuation (more than 152 HU, red areas) (**b**). The lesions above cutoff level (SUV > 4.0) were chosen (red lesions) (**c**). Each lesion’s TLG was calculated by the product of the volume and mean SUV of each bone lesion, and then, the summation of TLG values of whole skeletal lesions gave the WS-TLG value

Figure 1 shows the process to obtain active WS-TLG. These processes were performed semi-automatically by a software GI-BONE (AZE, Kanagawa, Japan).

### Deciding the threshold value

The maximum of Standardized Uptake Value (SUVmax) was measured in middle thoracic vertebra (Th7), 3rd lumbar vertebra (L3), sternum, rib, and ilium in 41 FDG–PET/CT studies in the breast cancer patients without skeletal metastasis. These regions were selected based on the frequency of skeletal metastasis [14]. A tentative threshold value was determined by the measurement of normal bone SUVmax distribution. Then, WS-TLG was calculated for 85 patients without skeletal metastasis and 35 patients with skeletal metastasis changing cutoff levels.

The negative rates, that is, any number above 0, in 85 patients without skeletal metastasis (specificity) and the positive rates in 35 patients with skeletal metastasis (sensitivity) were calculated. We decided a cutoff value by comparing specificity and sensitivity.

### Sensitivity and specificity

Sensitivity and specificity of WS-TLG were calculated using the cutoff level in 35 patients with newly developed skeletal metastasis and 85 patients without skeletal metastasis. In patients with skeletal metastasis, WS-TLG value differences were investigated based on CT types of skeletal metastasis.

### WS-TLG in patients with FDG follow-up

WS-TLG changes in each patient were compared with the results using PET Response Criteria in Solid Tumor (PERCIST) or European Organization for Research and Treatment of Cancer (EORTC) criteria [15–17].

We analyzed skeletal metastasis by PERCIST or EORTC regardless of other metastatic lesions. In PERCIST, up to two osseous lesions with highest FDG uptake (the peak standardized uptake value corrected for lean body mass: SULpeak) were chosen and followed on subsequent studies. When the therapeutic regimens changed, new sets of osseous lesions were selected.

When initial FDG uptake was low and the PERCIST could not be applied, EORTC criteria were applied to up to two osseous lesions regardless of other metastases. For EORTC, the sum of SUVmax was used, and for PERCIST, SULpeak values were used for evaluation [17]. Both analyses were performed by PET VCAR for Advantage Workstation (GE, USA).

The PERCIST metabolic response categories were complete metabolic response (CMR); no FDG-avid tumor visible (SULpeak was decreased to the background level), partial metabolic response (PMR); target lesions must show SULpeak decrease of greater than or equal to 30% and of at least 0.8 SUL units, progressive metabolic disease (PMD); SULpeak of target lesions must show an increase of greater than or equal to 30% and an increase of at least 0.8 SUL units or development of one or more new lesions; and stable metabolic disease (SMD); a response between PMR and PMD [18].

The EORTC response was divided into four categories: CMR was complete resolution of FDG uptake within all lesions, PMR was a reduction in the sum of SUVmax of at least 25% decrease after more than 1 treatment cycle, PMD was an increase of at least 25% in the sum of SUVmax or a new FDG-avid lesion, and SMD was response between PMR and PMD [17].

## Results

### Search for cutoff level

Table 1 shows the SUVmax values in various skeletal sites in 41 patients without skeletal metastasis. The SUVmax values were variables depending on sites. Among them, vertebral SUVmax values were higher than other sites. In vertebral sites, SUV max + 3SD was around 3.5. We regarded the SUV = 3.5 as the starting point to search for the optimal cutoff level.

**Table 1:** FDG SUVmax values in various bones in breast cancer patients without skeletal metastasis

Bone site	SUVmax value^a^				Mean + 2SD	Mean + 3SD
	Mean	SD	Minimum	Maximum		
Sternum	1.43	0.33	0.86	2.17	2.09	2.42
Rib	1.27	0.43	0.78	2.37	2.13	2.56
Th7	2.29	0.36	1.66	2.97	3.01	3.37
L3	2.33	0.36	1.56	3.26	3.05	3.41
Ilium	1.66	0.31	1.09	2.33	2.28	2.59

^a^Values were measured on FDG–PET/CT images in breast cancer patients without skeletal metastasis

SUVmax: maximum of standardized uptake value

Then, WS-TLG calculations were performed on 85 patients without and 35 patients with skeletal metastasis by changing cutoff levels (3.5, 4.0, and 4.5).

Table 2 shows WS-TLG specificities in patients without skeletal metastasis and sensitivities in skeletal metastasis with various cutoff levels (3.5, 4.0, and 4.5). Since SUV = 4.0 gave the specificity of 91% and the high sensitivity (97%) in patients with skeletal metastasis, we decided on SUV = 4.0 as the tentative cutoff level.

**Table 2:** WS-TLG values in breast cancer patients without or with bone metastasis with the function of cutoff value

		SUV^c^ Cutoff value		
		3.5	4.0	4.5
WS-TLG^a^ in patients without bone metastasis	Negative	69^b^/85	77/85	80/85
(85 patients)	Specificity (%)	81%	91%	94%
WS-TLG in patients with bone metastasis	Positive	34/35	34/35	32/35
(35 patients)	Sensitivity (%)	97%	97%	91%

^a^WS-TLG (whole skeletal total lesion glycolysis)

^b^Number of patients

^c^SUV (standardized uptake value)

## Sensitivity and specificity

The specificity was 91% (77/85) in the 85 breast cancer patients without skeletal metastasis.

The sensitivity was 97% (34/35) in 35 breast cancer patients with newly developed skeletal metastasis.

The WS-TLG values (mean ± SD) according to type of skeletal metastases (Table 3), were 30.9 ± 45.6 for the osteoblastic type, 30.9 ± 43.3 for the intertrabecular type (invisible type), 208.7 ± 326.3 for the osteolytic type, and 393.7 ± 680.0 for the mixed type, respectively. Statistical analysis was not performed because the number of patients was small. However, WS-TLG tended to be low in osteoblastic and intertrabecular types, and high in osteolytic and mixed types.

**Table 3:** WS-TLG values applying 4.0 cutoff value in breast cancer patients with newly diagnosed skeletal metastasis

CT type of bone metastasis	No. patients	WS-TLG^a^-positive	(%)	Mean WS-TLG	SD
Blastic type	5	5	100%	30.9	45.6
Intertrabecular type	6	5	83%	30.9	43.3
Lytic type	12	12	100%	208.7	326.3
Mixed type	12	12	100%	393.7	680.0
Total	35	34	97%	216.2	454.7

^a^WS-TLG; whole skeletal total lesion glycolysis

### Follow-up

WS-TLG in patients with skeletal metastasis who were followed up by repeated FDG study.

A total of 15 breast cancer patients with skeletal metastasis and pre-treatment FDG–PET/CT studies, and at least two follow-up FDG–PET/CT studies were selected randomly.

As shown in Table 4 (1, 2, and 3), 4 patients had favorable bone responses (WS-TLG) to therapy, 5 patients had progressive bone responses, and 6 patients had fluctuating responses.

**Table 4:** Comparison of WS-TLG and PERCIST or EORTC in breast cancer skeletal metastatic lesions; improved group

Case No.	FDG study^a^	Age	WS-TLG	PERCIST	EORTC	CT type		Comment
1	Pre Tx	49	11.6			Mixed		
	3M		0	CMR		Blastic		
	1Y 7M		0	CMR		Normal		
2	Pre Tx	70	0	NA		Intertrabecular		
	1Y 1M		0		SMD	Blastic		
	2Y 8M		0		SMD	Blastic		
	3Y 5M		0		SMD	Blastic		
3	Pre Tx	57	22.0			Mixed (lytic predominant)		
	4M		0.5	PMR		Blastic		
	8M		0	CMR		Blastic		
	1Y		0	CMR		Blastic		
4	Pre Tx	44	22.4			Blastic, faint		
	7M		0	CMR		Blastic, intense		
	1Y 7M		0	CMR		Blastic, intense		
	2Y		0	CMR		Blastic, intense		

*PERCIST *PET response criteria in solid tumor, *EORTC *criteria developed by the European Organization for Research and Treatment of Cancer, *Tx *treatment, *NA *not applicable, *CMR* complete metabolic remission, *PMR *partial metabolic response, *SMD *stable metabolic disease, *PMD* progressive metabolic disease, *WS-TLG *whole skeletal total lesion glycolysis

^a^Time after commencement of therapy for skeletal metastasis

In the favorable WS-TLG response group (Table 4), all 4 patients showed decreasing or very low at pre-therapy value (0) and no increase at follow-up studies. WS-TLG values and bone PERCIST or bone EORTC response were concordant.

**Fig. 2:**
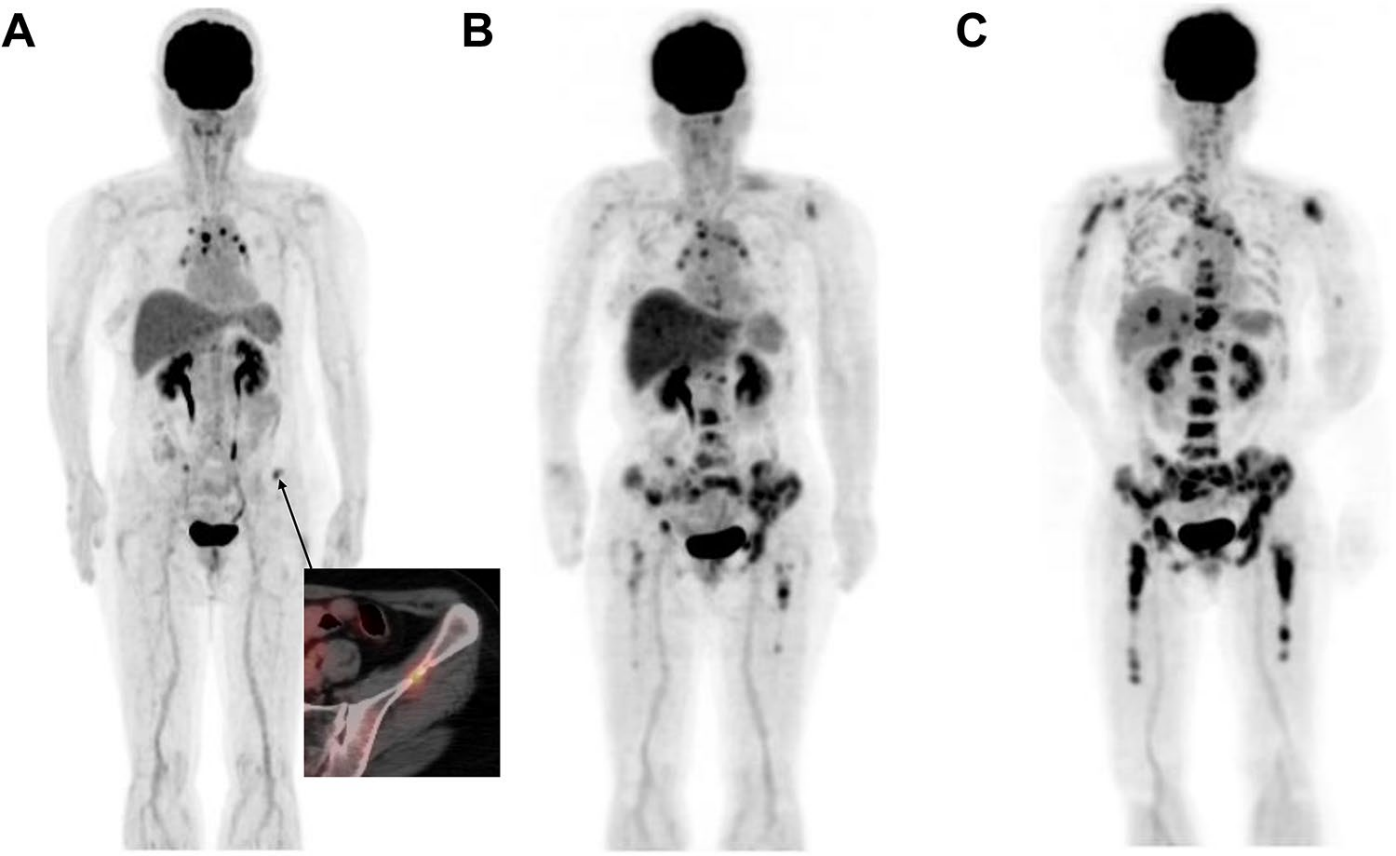
This is Case No. 9 in Table 5. Anterior views of FDG–PET MIP images at the time of diagnosis (**a**) and first (**b**), and second (**c**) follow-up studies are shown. She received FDG–PET/CT due to an elevation of a tumor marker (CA15-3) after her surgery of breast cancer. Oligo bone metastasis was noticed in bilateral iliac bones (**a**), and hormone therapy had begun. First follow-up FDG–PET/CT study showed an increased number of skeletal metastasis (**b**), then her skeletal metastasis further progressed at second follow-up (**c**). Her initial WS-TLG value was 0.2, and the value increased rapidly to 199.2 at first follow-up, and 889.4 at second follow-up

In the progressive WS-TLG response group (Table 5), WS-TLG changes and bone PERCIST or EORTC agreed in all 5 patients. A representative case (Case 9) is shown in Fig. 2. She underwent FDG–PET/CT due to an elevation of a tumor marker (CA15-3) after her surgery for breast cancer. Oligo bone metastases were noted in bilateral iliac bones (A), and her treatment was changed to hormone therapy. The first follow-up FDG–PET/CT study showed an increased number of skeletal metastases (B), and then her skeletal metastases progressed at the second follow-up (C).

**Table 5:** Comparison of WS-TLG and PERCIST or EORTC in breast cancer skeletal metastatic lesions; progressed group

Case No.	FDG study^a^	Age	WS-TLG	PERCIST	EORTC	CT type		Comment
5	Pre Tx	58	2.8			Blastic		
	1Y		76.7	PMD		Mixed		
	2Y		532.5	PMD		Mixed		
6	Pre Tx	57	0.4			Intertrabecular		
	9M		15.3	PMD		Mixed		
	1Y 6M		509.8	PMD		Mixed		
7	Pre Tx	65	0	NA		Intertrabecular		
	5M		6.6		PMD	Intertrabecular		
	11M		23.2		PMD	Intertrabecular		
8	Pre Tx	55	0	NA		Intertrabecular		
	10M		1.0		PMD	Blastic		
	1Y 7M		234.8		PMD	Blastic		
9	Pre Tx	71	0.2			Intertrabecular to lytic		
	1Y 5M		199.2	PMD		Blastic		
	1Y 9M		889.4	PMD		Blastic		

*PERCIST *PET response criteria in solid tumor, *EORTC *criteria developed by the European Organization for Research and Treatment of Cancer, *Tx *treatment, *NA *not applicable, *CMR *complete metabolic remission, *PMR *partial metabolic response, *SMD *stable metabolic disease, *PMD *progressive metabolic disease, *WS-TLG *whole skeletal total lesion glycolysis

^a^Time after commencement of therapy for skeletal metastasis

**Fig. 3:**
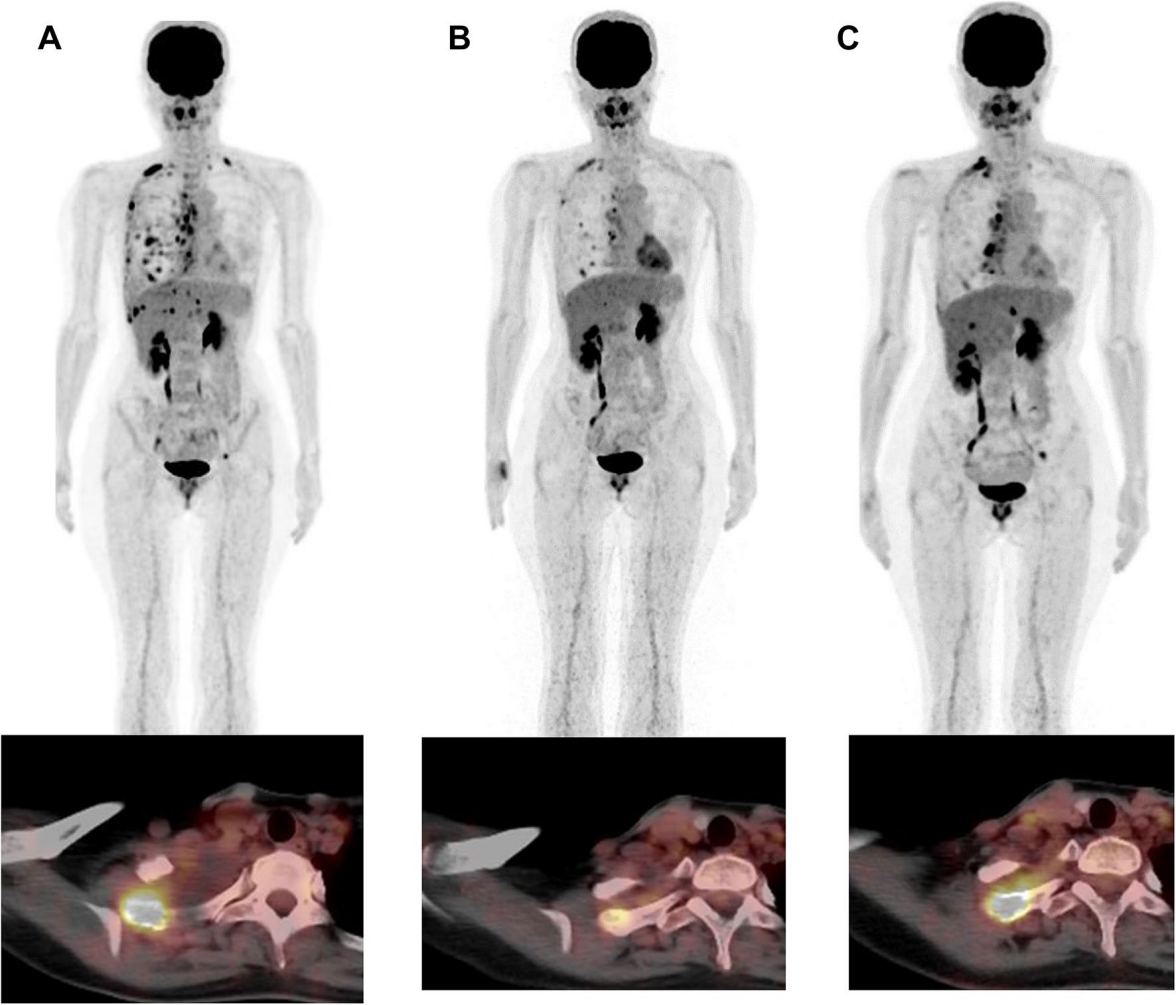
This is Case No. 11 in Table 6. Anterior views of FDG–PET MIP images, and FDG–PET and CT fusion axial images at the right first rib are shown. She noticed shortness of breath 10 years after her right breast surgery. FDG–PET/CT was performed, and pleural dissemination and multiple osteolytic skeletal metastases were diagnosed (**a**). She was prescribed hormone therapy after the FDG–PET/CT study (**a**). First follow-up FDG–PET/CT was performed after 6 months of hormone therapy and showed improvement in pleural dissemination and skeletal metastases (**b**). Because her tumor marker (CA15-3) was elevated (42.3 U/ml), second follow-up FDG–PET/CT was performed. FDG uptake in skeletal metastasis has increased and was judged to be progressive disease (**c**). Her initial WS-TLG value was 15.5, and the value was 0 at first follow-up and 4.6 at second follow-up FDG–PET/CT study

In fluctuating WS-TLG response group (Table 6), WS-TLG changes and bone PERCIST were concordant in all 6 patients. A representative case (Case 11) is shown in Fig. 3. She noticed shortness of breath 10 years after her right breast surgery. FDG–PET/CT was performed, and pleural dissemination and multiple osteolytic skeletal metastases were diagnosed (A). She was prescribed hormone therapy after the initial FDG–PET/CT study. The first follow-up FDG–PET/CT was performed after 6 months of hormone therapy and showed improvement in pleural dissemination and skeletal metastases (B). Since her tumor marker (CA15-3) was elevated (42.3 U/mL), a second follow-up FDG–PET/CT was performed. FDG uptake in skeletal metastasis had increased and was judged to be progressive disease (C).

**Table 6:** Comparison of WS-TLG and PERCIST or EORTC in breast cancer skeletal metastatic lesions; fluctuated group

Case No.	FDG study^a^	age	WS-TLG	PERCIST bone	EORTC bone	CT type		Comment
10	Pre Tx	61	254.7			Intertrabecular		
	4M		43.5	PMR		Blastic		
	9M		107.1	PMD		Blastic		
11	Pre Tx	38	15.5			Lytic		
	6M		0	PMR		Mixed		
	1Y 1M		4.6	PMD new lesion		Lytic, increased		
12	Pre Tx	67	13.6			Mixed		
	6M		0	CMR		Blastic		
	1Y		0	CMR		Blastic		
	1Y 6M (pre-Tx)		0	CMR		Blastic		Tx regimen change
	(4M)		1.0	PMD new lesion		Mixed: blastic and intertrabecular		
13	Pre Tx	59	73.4			Mixed		
	10M (pre-Tx)		80.2	SMD		Mixed		Tx regimen change
	(1Y)		0.4	CMR		Blastic		
14	Pre Tx	60	24.5			Intertrabecular		
	8M		0	CMR		Mixed; intertrabecular and lytic		
	11M		0	PMR		Blastic		
	1Y 5M (pre Tx)		3.8	PMD		Blastic		Tx regimen change
	(1Y)		12.8	PMD		Blastic		
15	Pre Tx	65	13.3			Lytic		
	1Y (pre Tx)		72.4	PMD		Mixed		Tx regimen change
	(4M)		0.1	PMR		Mixed		
	(9M)		10.2	PMD		Mixed		
	(1Y)		14.1	SMD		Mixed		

*PERCIST *PET response criteria in solid tumor, *EORTC *criteria developed by European Organization for Research and Treatment of Cancer, *Tx* treatment, *CMR* complete metabolic remission, *PMR* partial metabolic response, *SMD* stable metabolic disease, *PMD* progressive metabolic disease, *WS-TLG* whole skeletal total lesion glycolysis

^a^Time after commencement of treatment for skeletal metastasis. () means time after the change of therapy

## Discussion

The bone is the most common site to which breast cancer metastasizes. Assessment of the change in tumor burden is an important feature of clinical evaluation of cancer therapeutics. However, oncologists often find difficulty in evaluating the therapeutic effect on skeletal metastasis. Historically, assessment methods of tumor response criteria developed by International Union Against Cancer (UICC) and World Health Organization (WHO) have been used [2]. They were based on plain x-ray and bone scintigraphy. Recent criteria developed by the Response Criteria in Solid Tumors (RECIST 1.1) stated that (1) Bone scan, PET scan, or plain films are not considered adequate imaging techniques to measure bone lesions. (2) Lytic bone lesions or mixed lytic-blastic lesions, with identifiable soft tissue components, that can be evaluated by cross sectional imaging techniques such as CT or MRI can be considered as measurable lesions if the soft tissue component meets the definition of measurability. (3) Blastic bone lesions are non-measurable [18]. This means that most skeletal metastases cannot be evaluated for therapeutic response. A group from MD Anderson Hospital proposed revised criteria for bone response [2, 19]. Other criteria are PERCIST and EORTC, in which tumor response is measured by FDG–PET uptake. Both criteria measure the change in FDG uptake in many involved organ lesions, not restricted to bone lesions. The existing criteria all use the classification of complete (metabolic) response, partial (metabolic) response, stable (metabolic), and progressive (metabolic) disease.

Apart from the response classification, we intended to make a continuous biomarker of bone tumor burden using FDG–PET/CT. We here propose a biomarker; a summation of TLGs of active bone lesions above the cutoff level. This WS-TLG method shows the glucose uptake activity in the whole skeletal lesion above threshold as a continuous number (a summation of the product of lesion volume and SUVmean of each bone lesion). We imagined this WS-TLG value may be used, for example, serum alkaline phosphatase value or carcinoembryonic antigen level.

The cutoff level was decided using 85 patients without skeletal metastasis. We tentatively decided on SUV = 4.0 as the cutoff value, which gave 91% of specificity (77/85) and 97% of sensitivity (34/35). Of course, cutoff value will change the study population, and this value is only a tentative one.

The WS-TLG values in patients with newly developed skeletal metastasis varied among CT types. Osteoblastic and intertrabecular types showed low values, and lytic and mixed types showed high values; no statistical evaluation could be performed because the number of patients was small. The low WS-TLG of blastic type may be the reflection of low FDG avidity of blastic metastasis [20]. The low WS-TLG of the intertrabecular type may reflect that intertrabecular type is the beginning of skeletal metastasis. High values in lytic and mixed types may indicate that this method is suitable for these CT types of skeletal metastasis.

In this study, we compared WS-TLG with PERCIST or EORTC for bone metastasis. Very good agreement was achieved in all 15 patients.

The limitations of this study are that this is the first study of WS-TLG regarding osseous metastases from cancer. WS-TLG only gives the quantitative FDG activity restricted to bone area regardless of other organs. That is, when a patient who is receiving chemotherapy for metastatic bone lesions shows improvement in bone lesions but other new (for example, new pulmonary metastasis) lesions develop, the patient’s WS-TLG will improve but the total judgement of the chemotherapeutic effect is progression of disease due to the development of a new pulmonary lesion. This point can be interpreted as both an advantage (this method can evaluate bone lesions separately) and disadvantage (this method cannot evaluate other organs).

Three patients (one in the improved group and two in the progressed group) in follow-up showed WS-TLG = 0 at the initial assessment. The percentage of FDG non-avid skeletal metastasis differed between newly diagnosed 35 patients (1/35) and follow-up 15 patients (3/15). We thought that FDG non-avid skeletal metastasis might be inadequate to evaluate by WS-TLG; therefore, we intentionally included FDG non-avid patients in the follow-up group. Although 3 patients whose skeletal lesions were FDG non-avid (WS-TLG = 0) were successfully evaluated, there is still the possibility that FDG study including WS-TLG is not suitable to evaluate the therapeutic effect or clinical response in FDG non-avid skeletal lesions.

Another limitation is different performance of each PET/CT machine. Since SUV values obtained by old PET/CT machines differ from those by new machines and WS-TLG is calculated based on SUV value, we cannot directly compare the WS-TLG values between old and new PET/CT machines. We currently use three different PET/CT machines. Phantom studies showed that SUVs obtained by an old PET/CT machine (Canon Aquiduo) were about 20–30% (depending on the lesion size) lower than those obtained by new machines (Discovery 610 and IQ). The SUVs of Discovery 610 and Discovery IQ were equivalent. Therefore, we excluded the data obtained by Aquiduo PET/CT machine.

This study was only a single-institute retrospective study. The number of patients was not enough, and the interval between FDG studies was not fixed. There is no gold standard to evaluate skeletal metastases; therefore, we employed PERCIST or EORTC for bone as the comparative standard. As mentioned earlier, it is unclear whether this WS-TLG method can be applied to non-FDG avid skeletal metastasis.

This WS-TLG method consisted of automated organ (bone) segmentation from CT images and extraction of FDG-avid lesions using a cutoff level. Automated organ segmentation or extraction by CT images is now progressing rapidly [21]. Soon, FDG uptake of each organ (e.g., lung, liver, or etc.) can be separately evaluated. We hope that our study of WS-TLG is the beginning of organ extraction and tracer uptake evaluation in a clinical setting.

## Conclusion

We have developed a semi-automatic quantitative method (WS-TLG) to evaluate skeletal metastases using FDG–PET/CT data. The cutoff value was decided using FDG–PET/CT data of breast cancer patients with and without skeletal metastasis, and SUV = 4.0 was the tentative cutoff value. Using the cutoff value, 97% (34/35) sensitivity and 91% (77/85) specificity were obtained. The WS-TLG method was applied to 15 breast cancer patients with skeletal metastases who underwent therapy. WS-TLG was compared with PERCIST or EORTC only for bone, and very good agreement (15/15) was obtained. This quantitative WS-TLG method seems to be a good biomarker to evaluate skeletal metastasis in breast cancer patients. Further clinical studies are warranted to evaluate the clinical values of this new WS-TLG method.

## Conflict of interest

All authors have no potential conflicts of interest to disclose.
